# Securing the Airway in a Patient With a Penetrating Neck Injury: A Case Report

**DOI:** 10.7759/cureus.96631

**Published:** 2025-11-11

**Authors:** Sanjay K Meena, Manish Garg, Chhavi Sawhney, Abhishek Singh

**Affiliations:** 1 Anesthesiology, All India Institute of Medical Sciences, New Delhi, New Delhi, IND

**Keywords:** difficult airway management, fiberoptic bronchoscopy, neck trauma, penetrating neck injury, videolaryngoscope

## Abstract

Penetrating neck injury presents significant challenges for anesthesiologists due to distorted anatomy, bleeding, and the risk of sudden deterioration. Airway management requires careful consideration of the technique selection to balance urgency with patient safety.

We report a 30-year-old male with a self-inflicted knife injury to the anterior neck. Imaging revealed the blade traversing the sternohyoid muscle, grazing the thyroid, and extending toward the apical pleura, without vascular or airway breach. The patient was hemodynamically stable on arrival. After multidisciplinary planning, anesthesia was induced using a modified rapid sequence induction. Endotracheal intubation was successfully achieved on the first attempt with a video laryngoscope. A fiberoptic bronchoscope was introduced post-intubation to exclude airway injury. The knife was removed surgically without complications. Repeat bronchoscopy prior to extubation confirmed airway integrity. The perioperative course was uneventful, and the patient was discharged after one week with no sequelae. This case highlights the value of a combined approach using video laryngoscopy for primary airway control and post-intubation fiberoptic bronchoscopy for confirmation of tube placement and evaluation of internal injury. In penetrating neck trauma, such an integrated strategy enhances airway safety while minimizing additional harm. Nevertheless, preparedness for an immediate surgical airway remains essential, given the potential for rapid clinical deterioration.

## Introduction

Penetrating neck injuries (PNIs) represent one of the most challenging emergencies in trauma care, accounting for approximately 5%-10% of all traumatic injuries [1]. The anatomical complexity of the neck, with its dense concentration of vital structures, including the trachea, larynx, major blood vessels, esophagus, and cervical spine within a confined space, makes these injuries particularly hazardous. The mortality rate associated with PNI can range from 3% to 10%, with airway compromise being a leading cause of preventable death [2].

The neck is anatomically divided into three zones for trauma assessment purposes. Zone I extends from the clavicle to the cricoid cartilage, Zone II from the cricoid cartilage to the angle of the mandible, and Zone III from the angle of the mandible to the skull base [3]. Zone II injuries, which encompass the majority of penetrating neck trauma cases, pose particular challenges for airway management due to the proximity of the larynx, trachea, and major vascular structures.

For anesthesiologists, the management of PNI presents a complex decision-making scenario. The primary concern is securing a patent airway while minimizing the risk of exacerbating existing injuries or creating new ones [4]. Traditional airway management approaches may be contraindicated or carry increased risks in the presence of anatomical distortion, active bleeding, hematoma formation, or potential vascular injury [4]. The choice between awake versus asleep intubation, the selection of intubation technique, and the decision regarding surgical airway creation must be individualized based on patient presentation, injury characteristics, and available resources [5].

The literature reveals varying approaches to airway management in PNI. Awake fiberoptic intubation (AFI) has traditionally been considered the gold standard for anticipated difficult airways, particularly when airway anatomy is distorted or when there is concern for airway disruption [6]. This technique allows for preservation of spontaneous ventilation and provides the ability to assess airway patency during the procedure. However, practical limitations, including patient cooperation, time constraints, equipment availability, and operator expertise, may preclude its use in emergency situations [7].

Alternatively, controlled rapid sequence induction (RSI) with video laryngoscopy (VL) can be a viable option for hemodynamically stable patients with PNI [8,9]. VL offers improved glottic visualization compared to direct laryngoscopy (DL) and may reduce the degree of neck manipulation required for successful intubation [10]. The technique has demonstrated high success rates in trauma patients and may be particularly beneficial when awake intubation is not feasible [10].

Surgical airway management, including cricothyroidotomy and tracheostomy, remains the ultimate backup plan for failed intubation scenarios [11]. The decision to proceed directly to surgical airway creation may be necessary in cases of severe airway distortion, complete airway obstruction, or when conventional intubation techniques have failed [12]. Recent guidelines emphasize the importance of early consideration and preparation for surgical airway management in PNI cases [13].

We present a case that exemplifies the successful management of a patient with PNI, highlighting the decision-making process, technical considerations, and multidisciplinary approach required for safe airway management in this challenging clinical scenario.

## Case presentation

**Figure 1:**
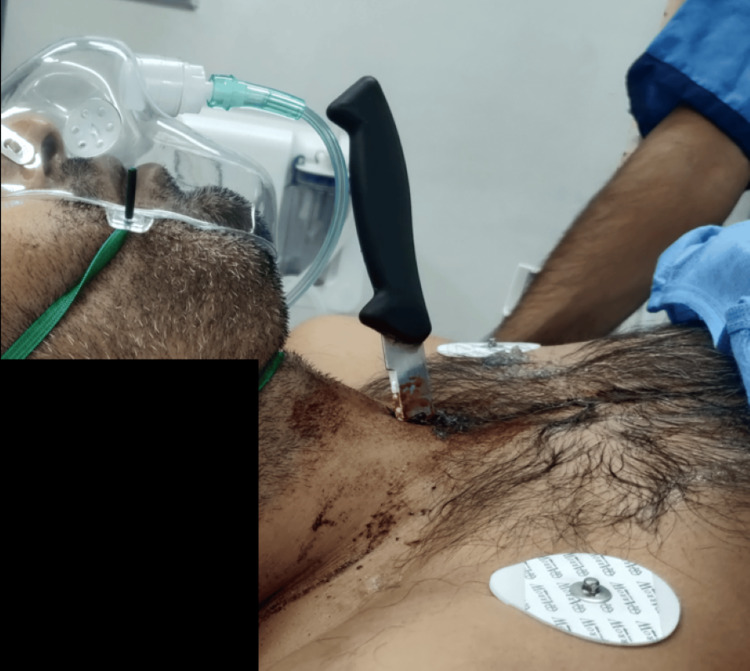
Clinical image showing a knife lodged in the anterior neck at presentation.

A 30-year-old male, classified as American Society of Anesthesiologists (ASA) physical status II and with a body mass index (BMI) of 24 kg/m², presented to the Emergency Department with a self-inflicted stab wound to the anterior aspect of the neck (Figure 1). 

**Figure 2:**
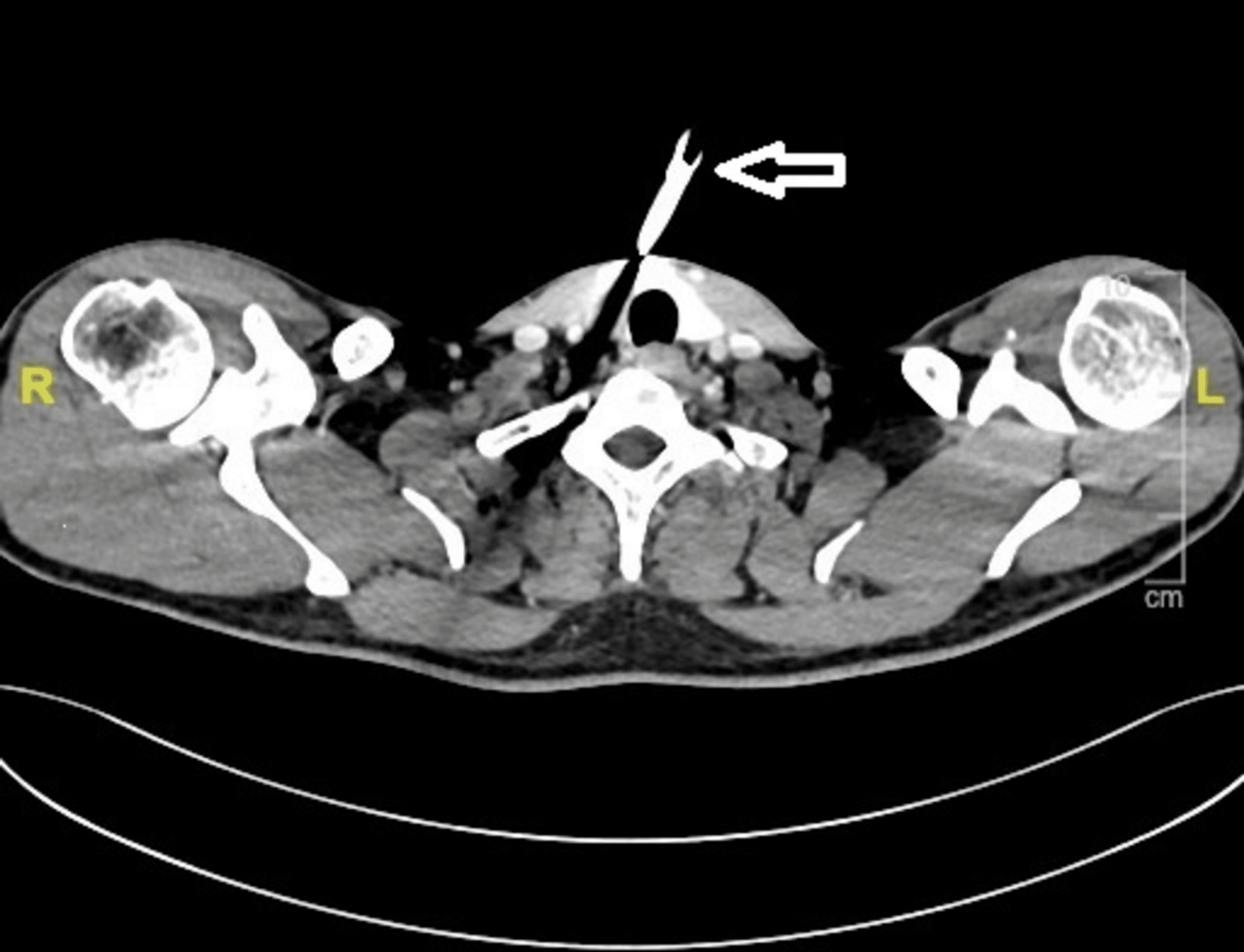
CT image depicting the knife trajectory (white arrow) through the sternohyoid muscle and thyroid gland, extending toward the apical pleura, and sparing major vascular structures. CT, computed tomography

On initial assessment, the patient was supine, conscious, and oriented, with stable vital signs (heart rate 88/min and blood pressure 126/86 mmHg) but was uncooperative. There was no history of loss of consciousness, vomiting, seizures, dyspnea, stridor, hemoptysis, or subcutaneous emphysema around the neck following the injury. Radiological imaging demonstrated a metallic knife traveling from the midline to the right lateral aspect of the neck, piercing the sternohyoid muscle and grazing the inferior pole of the thyroid gland. The trajectory extended toward the right apical pleura but spared the carotid and subclavian vessels. No evidence of airway breach was noted (Figure 2). 

A comprehensive airway assessment was performed to evaluate potential difficulties in intubation and ventilation. The patient's mouth opening was adequate (>3 finger breadths), with a Mallampati Class II view. The thyromental distance was measured at 7 cm, and the neck had a normal range of motion prior to injury. There were no signs of micrognathia, macroglossia, or dental abnormalities. There was no history of obstructive sleep apnea or previous difficult intubation. Based on these findings, apart from the anatomical distortion caused by the embedded knife, no additional predictors of difficult intubation or ventilation were identified.

A multidisciplinary airway management plan was formulated in consultation with the trauma surgery team. The primary plan was a modified RSI with VL to minimize aspiration risk while ensuring optimal visualization. The backup plan included AFI if the primary approach appeared unsafe, and an immediate surgical airway (cricothyroidotomy or tracheostomy) as the rescue technique if both noninvasive methods failed. The trauma surgeon was present in the operating room throughout the procedure, with surgical instruments prepared for emergency tracheostomy if required. A difficult airway cart was positioned at the bedside, and a cricothyroidotomy kit was opened and kept ready for immediate use.

**Figure 3:**
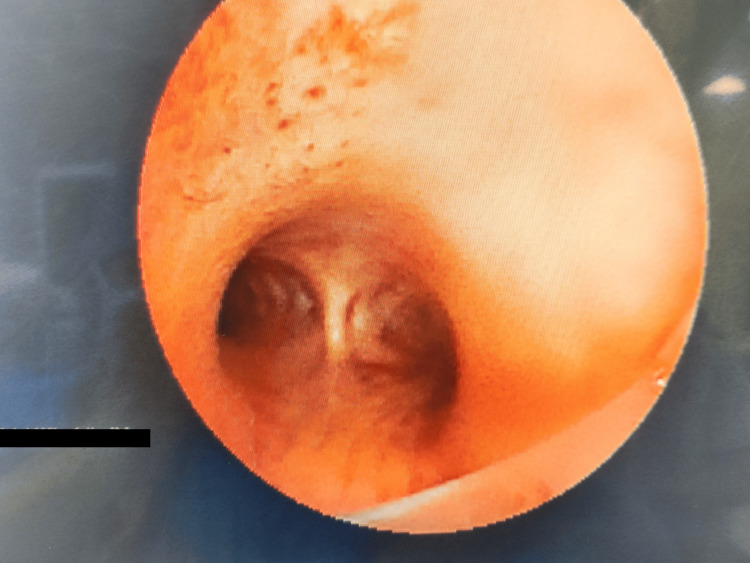
Fiberoptic bronchoscopy image confirming intact airway with no injury visualized up to the level of the carina.

Standard ASA monitoring was established, including continuous electrocardiography, pulse oximetry, noninvasive blood pressure monitoring, capnography, and temperature monitoring. Preoxygenation was carefully performed via facemask with 100% oxygen for three minutes, without applying pressure to avoid displacement of the knife or worsening of the injury, achieving an end-tidal oxygen concentration above 90%. Anesthesia was induced using a modified RSI with intravenous fentanyl 2 mcg/kg, propofol 2 mg/kg, and rocuronium 1.2 mg/kg, after confirming adequate mask ventilation. Intubation was achieved successfully on the first attempt using a D-blade of the C-MAC® video laryngoscope (Karl Storz SE & Co. KG, Tuttlingen, Germany) with an 8.0 mm endotracheal tube (ETT). Dexamethasone 8 mg was administered intravenously immediately after induction to minimize airway edema and reduce postoperative nausea and vomiting. Anesthesia was maintained with sevoflurane in an oxygen-air mixture, with a minimum alveolar concentration (MAC) of 0.8-1.0. A flexible fiberoptic bronchoscope was subsequently introduced through the ETT, confirming the absence of any airway injury up to the carina (Figure 3).

The surgical procedure involved the removal of the knife under direct visualization. Intraoperative analgesia was provided with incremental doses of fentanyl. Additional analgesia included intravenous paracetamol 1 g, administered 30 minutes before the end of surgery. Local infiltration with 10 mL of 0.25% bupivacaine was performed by the surgical team at the wound site before closure. Prior to extubation, a repeat fiberoptic bronchoscopy (FB) was performed, which confirmed airway integrity.

The extubation strategy was carefully planned to minimize the risk of coughing and bucking, which could potentially disrupt the surgical repair or cause bleeding at the wound site. An awake extubation technique was selected over deep extubation to ensure adequate respiratory drive and the ability to protect the airway in case of postoperative bleeding or edema. To prevent vigorous coughing during emergence, intravenous lignocaine 1.5 mg/kg was administered 90 seconds before extubation to suppress airway reflexes. The oropharynx was gently suctioned under direct vision to remove any secretions without stimulating the posterior pharyngeal wall. Sevoflurane concentration was gradually reduced, while maintaining adequate depth of anesthesia, to allow smooth emergence. Once the patient demonstrated adequate spontaneous ventilation, purposeful movements, and the ability to follow commands, neuromuscular blockade was reversed, and the ETT was removed gently at the end of a normal exhalation while the patient was breathing 100% oxygen. Throughout the extubation process, the patient remained calm, without any episodes of coughing, straining, or hemodynamic instability. Postoperative analgesia was managed with intravenous paracetamol 1 g every six hours, and tramadol 50 mg intravenously as needed for breakthrough pain. The postoperative course was uneventful, and the patient was discharged after one week without any complications.

## Discussion

PNI represents one of the most challenging scenarios in trauma anesthesia, requiring rapid decision-making in the face of potentially life-threatening airway compromise. The anatomical complexity of the neck, with its concentration of vital structures within a small space, creates unique challenges that differ significantly from routine airway management [4]. The present case illustrates several key principles and considerations that are fundamental to successful outcomes in these high-risk situations.

Patient assessment and risk stratification

The initial assessment of patients with PNI must balance thoroughness with urgency. In our case, the patient's hemodynamic stability and absence of respiratory distress allowed for a systematic evaluation approach. However, the clinical presentation can change rapidly, making continuous reassessment essential [4,5,8]. The absence of stridor, subcutaneous emphysema, hemoptysis, or voice changes in our patient was reassuring, but these signs may be absent even in the presence of significant airway injury [4].

The role of imaging in penetrating neck trauma remains controversial, with some advocating for immediate surgical exploration while others support selective imaging-based management [2]. In our case, computed tomography provided valuable information about the trajectory of the knife and helped identify structures at risk. However, imaging should never delay definitive airway management when immediate intervention is required [2]. The decision to obtain imaging must be individualized based on patient stability, mechanism of injury, and clinical findings.

Airway management strategy selection

The choice of airway management technique in PNI has evolved significantly over the past decade. Traditionally, AFI was considered the standard approach due to its ability to maintain spontaneous ventilation and provide real-time airway assessment [8,9]. However, this technique requires patient cooperation, adequate topical anesthesia, and considerable expertise - factors that may not be readily available in emergency situations [10].

Recent evidence suggests that RSI with VL can be equally effective in appropriately selected patients [14-16]. The advantages of this approach include faster execution, reduced patient discomfort, and the elimination of the need for patient cooperation. In our case, the patient was hemodynamically stable, exhibited no signs of respiratory compromise, and had a normal airway on initial evaluation. These factors supported the decision to proceed with an RSI instead of awake intubation. Cricoid pressure was intentionally avoided, as any manipulation could have displaced the penetrating object and potentially exacerbated the underlying injury.

The selection of VL over DL offers several theoretical advantages in PNI. VL provides superior glottic visualization, can be performed in the neutral neck position, and may decrease the force applied during laryngoscopy [14-16]. These factors are particularly relevant when anatomical distortion or cervical spine injury is a concern. The use of a D-blade in our case facilitated successful intubation, with minimal neck manipulation.

Role of fiberoptic bronchoscopy

One of the most significant aspects of our case management was the systematic use of FB, both immediately after intubation and prior to extubation. This approach serves multiple purposes in PNI management. Post-intubation bronchoscopy allows for comprehensive airway inspection to identify occult injuries that may not be apparent on clinical examination or imaging studies. Even when the penetrating object appears to spare the airway based on trajectory analysis, microscopic injuries or mucosal disruption may be present.

The detection of airway injuries has important implications for ongoing management. Unrecognized tracheal or laryngeal injuries can lead to delayed complications, including airway obstruction, pneumothorax, pneumomediastinum, or surgical emphysema [17]. Early identification allows for appropriate surgical intervention and may prevent the need for emergency airway procedures during the postoperative period.

Pre-extubation bronchoscopy serves as a final safety check to ensure that surgical manipulation has not created new airway injuries and that the airway remains patent for safe extubation. This is particularly important in cases where surgical exploration occurs in close proximity to airway structures, as was the situation in our patient.

Multidisciplinary coordination

The successful management of PNI requires seamless coordination between multiple specialties, including anesthesiology, trauma surgery, otolaryngology, and, potentially, vascular surgery. In our case, the presence of the trauma surgeon during the intubation process allowed for immediate surgical backup if airway rescue became necessary. This "double setup" approach, where both medical and surgical airway options are immediately available, represents best practice in managing these complex cases [4].

Communication between team members must be clear and continuous, with predefined roles and escalation plans. The anesthesiologist should maintain primary responsibility for airway management while keeping surgical colleagues informed of the plan and any concerns that arise during the procedure. Having all necessary equipment immediately available, including emergency cricothyrotomy kits, is essential for rapid response to complications.

Technical considerations and equipment

The success of our airway management strategy was facilitated by the availability of advanced airway equipment and the team's familiarity with its use. VL has become increasingly prevalent in emergency airway management, but proficiency with the equipment is essential for optimal outcomes [14-16]. Regular training and simulation exercises help maintain skills and ensure that equipment functions properly when needed in emergency situations.

The choice of ETT size and type may also influence success rates in penetrating neck trauma. In our case, an 8.0 mm tube was selected to facilitate subsequent bronchoscopy, while providing adequate ventilation. However, in cases where airway edema or anatomical distortion is present, smaller tube sizes may be necessary.

Alternative approaches and backup plans

While our case proceeded smoothly with the chosen strategy, it is important to consider alternative approaches that might have been employed. AFI remains a viable option, particularly in cases where patient cooperation can be obtained and when concerns about airway anatomy are significant. The technique may be particularly valuable when the trajectory of the penetrating object suggests possible airway involvement, or when clinical signs of airway compromise are present.

Surgical airway management, including cricothyrotomy or emergency tracheostomy, must always be considered as a backup option. In cases where intubation attempts fail or where complete airway obstruction is present, immediate surgical airway creation may be life-saving. The decision to proceed directly to surgical airway management may also be appropriate in cases of severe anatomical distortion or when multiple failed intubation attempts have occurred.

Postoperative mechanical ventilation (POMV)

The decision regarding POMV in PNI requires careful consideration of multiple factors. While our patient was successfully extubated immediately after surgery, following confirmatory bronchoscopy, some cases may benefit from a period of POMV. Indications for POMV include significant airway edema from manipulation, extensive surgical dissection near airway structures, concerns about hemodynamic stability, or uncertainty regarding complete hemostasis. Prolonged mechanical ventilation allows time for resolution of tissue edema, stabilization of repaired structures, and ensures a secure airway during the critical early postoperative period, when complications are most likely to manifest. The decision must be individualized based on the extent of injury, complexity of surgical repair, the patient's overall condition, and available monitoring capabilities. In cases where extubation is performed, it should ideally occur in a controlled environment, with immediate access to reintubation equipment and personnel skilled in difficult airway management, as reintubation in the setting of postoperative edema and surgical changes can be significantly more challenging than the initial intubation.

## Conclusions

This case illustrates the successful management of a complex PNI through meticulous planning, appropriate technique selection, and effective multidisciplinary coordination. The combined use of VL for airway control and FB for post-intubation assessment offers a balanced approach that enhances both safety and efficiency in the emergency setting. It underscores the importance of individualized assessment, readiness with multiple airway options, and close anesthesiologist-surgeon collaboration. While AFI remains the gold standard for anticipated difficult airways, this case supports the selective use of modified RSI with VL in hemodynamically stable patients. Routine post-intubation bronchoscopy in PNI not only confirms airway integrity but may also detect occult injuries. Ultimately, successful PNI management relies on preparation, teamwork, and adaptive decision-making.
